# Oxaliplatin-Based Chemotherapy Is More Beneficial in *KRAS* Mutant than in *KRAS* Wild-Type Metastatic Colorectal Cancer Patients

**DOI:** 10.1371/journal.pone.0086789

**Published:** 2014-02-04

**Authors:** Yu-Lin Lin, Yi-Hsin Liang, Jia-Huei Tsai, Jau-Yu Liau, Jin-Tung Liang, Been-Ren Lin, Ji-Shiang Hung, Liang-In Lin, Li-Hui Tseng, Yih-Leong Chang, Kun-Huei Yeh, Ann-Lii Cheng

**Affiliations:** 1 Department of Oncology, National Taiwan University Hospital, Taipei, Taiwan; 2 Department of Pathology, National Taiwan University Hospital, Taipei, Taiwan; 3 Division of Colorectal Surgery, National Taiwan University Hospital, Taipei, Taiwan; 4 Department of Surgery, National Taiwan University Hospital, Taipei, Taiwan; 5 Department of Medical Research, National Taiwan University Hospital, Taipei, Taiwan; 6 Department of Laboratory Medicine, National Taiwan University Hospital, Taipei, Taiwan; 7 Department of Medical Genetics, National Taiwan University Hospital, Taipei, Taiwan; 8 Department of Internal Medicine, National Taiwan University Hospital, Taipei, Taiwan; 9 Department of Clinical Laboratory Sciences and Medical Biotechnology, National Taiwan University College of Medicine, Taipei, Taiwan; 10 Graduate Institute of Oncology, National Taiwan University College of Medicine, Taipei, Taiwan; 11 Graduate Institute of Clinical Medicine, National Taiwan University College of Medicine, Taipei, Taiwan; 12 Graduate Institute of Pathology, National Taiwan University College of Medicine, Taipei, Taiwan; 13 Department of Hemato-Oncology, E-da Hospital, Kaohsiung City, Taiwan; UT MD Anderson Cancer Center, United States of America

## Abstract

To identify better regimens in currently available chemotherapy would be beneficial to *KRAS* mutant metastatic colorectal cancer (mCRC) patients because they have fewer treatment options than *KRAS* wild-type mCRC patients. Clinicopathologic features and overall survival (OS) of *KRAS* mutant and wild-type mCRC patients who had used oxaliplatin-based, irinotecan-based, bevacizumab-based, as well as cetuximab-based regimens were compared to those who had never-used oxaliplatin-based, irinotecan-based, bevacizumab-based, as well as cetuximab-based regimens respectively. Between 2007 and 2012, a total of 394 mCRC patients, in whom 169 *KRAS* mutant and 225 *KRAS* wild-type, were enrolled. In *KRAS* mutant patients who had used oxaliplatin-based regimens (N = 131), the OS was significantly longer than that in *KRAS* mutant patients who had never-used oxaliplatin-based regimens (N = 38). The OS was 28.8 months [95% confidence interval (CI): 23.2–34.4] in *KRAS* mutant patients who had used oxaliplatin-based regimens versus 17.8 months [95% CI: 6.5–29.1] in *KRAS* mutant patients who had never-used oxaliplatin-based regimens (P = 0.026). Notably, OS in *KRAS* wild-type mCRC patients who had used oxaliplatin-based regimens (N = 185) was not significantly longer than that in *KRAS* wild-type mCRC patients who had never-used oxaliplatin-based regimens (N = 40) (P = 0.25). Furthermore, the OS in *KRAS* mutant patients who had used either irinotecan-based, bevacizumab-based or cetuximab-based regimens was not significantly different than that in *KRAS* mutant patients who had never-used either irinotecan-based, bevacizumab-based or cetuximab-based regimens respectively. In multivariate analyses, patients who had used oxaliplatin-based regimens remains an independent prognostic factor for longer OS in *KRAS* mutant mCRC patients. In conclusion, oxaliplatin-based regimens are more beneficial in *KRAS* mutant than in *KRAS* wild-type mCRC patients.

## Introduction

To identify potentially better regimens in currently available systemic chemotherapy, if existed, would be crucial to *KRAS* mutant metastatic colorectal cancer (mCRC) patients because they do not benefit from epidermal growth factor receptor (EGFR) monoclonal antibody and have fewer treatment options than *KRAS* wild-type mCRC patients. Mutation of the *KRAS* gene in mCRC has been identified as a negative predictor to EGFR monoclonal antibody [1]. Prospective randomized clinical trials have further demonstrated this finding [2]–[4]. In patients who received first-line chemotherapy plus EGFR monoclonal antibodies, progression-free survival (PFS) in *KRAS* mutant mCRC patients was shorter than that in *KRAS* wild-type patients. The PFS was 7.6 months in *KRAS* mutant group and 9.9 months in *KRAS* wild-type group in the CRYSTAL study [2]; 5.5 months in *KRAS* mutant group and 7.7 months in *KRAS* wild-type group in the OPUS study [3]; and 7.3 months in *KRAS* mutant group and 9.6 months in *KRAS* wild-type group in the PRIME [4] study. Based on the results of these randomized clinical studies, *KRAS* mutant mCRC patients have no longer been suggested to use EGFR monoclonal antibody [5]. Therefore, to identify potentially better regimens from currently available systemic treatments or to explore newer agents for the treatment of *KRAS* mutant mCRC patients is thus warranted.

Based on the subgroup analyses of the OPUS and PRIME studies, we previously conducted *in-vitro* [6] and retrospective clinical [7] proof-of-concept studies which demonstrated that *KRAS* mutant mCRC patients might benefit more from oxaliplatin-based chemotherapy than *KRAS* wild-type mCRC patients. In these recently published studies, we demonstrated that *KRAS* gene mutation in mCRC might be a predictor of oxaliplatin sensitivity not only in colon cancer cells but also in mCRC patients. In the *in-vitro* [6] study, we firstly demonstrated that *KRAS* mutant colon cancer cells are more sensitive to oxaliplatin than the same *KRAS* mutant colon cancer cells in which the expression of mutant *KRAS* was knocked down by small interfering RNA (siRNA). The mechanism of the sensitivity of oxaliplatin was through down-regulation of excision repair cross-complementation group 1 (ERCC1) which was a predictor of oxaliplatin resistance. We also found that resistant predictors of fluorouracil and irinotecan which were thymidylate synthase and topoisomerase I, respectively, were both unchanged in our experimental system. Additionally, in our retrospective clinical study [7], we further unveiled that *KRAS* mutant mCRC patients may have significantly longer first-line PFS than *KRAS* wild-type mCRC patients when both of them had received first-line oxaliplatin-based chemotherapy. In addition, *KRAS* gene mutation was an independent predictive factor for longer first-line PFS which was demonstrated not only in univariate but also in multivariate analyses in our study. Although growing evidence shows that oxaliplatin-based regimens might be a better chemotherapeutic partner in *KRAS* mutant mCRC, clinical studies enrolling more mCRC patients to investigate this issue from different research point-of-view remain warranted.

Our previous work inspired us to further investigate the potential benefit of oxaliplatin for *KRAS* mutant mCRC patients. We hypothesized that oxaliplatin benefits *KRAS* mutant mCRC patients more than it does for *KRAS* wild-type mCRC patients. This time, we approached this question through another gold standard end-point, OS. We tried to evaluate the potential OS difference between all *KRAS* mutant mCRC patients who had used and never-used oxaliplatin-based regimens to demonstrate how crucial oxaliplatin would be in *KRAS* mutant mCRC patients. However, what could be argued under this study design would be unavoidable selection bias and the dogma that more drugs always result in longer OS. To eliminate the influences of selection bias, we simultaneously compared the potential OS difference between mCRC patients who had used and never-used either oxaliplatin-based, irinotecan-based, bevacizumab-based, or cetuximab-based regimens in *KRAS* mutant and wild-type patients to delineate that our study results would not just merely caused by selection bias or the dogma that more drugs always result in longer OS. We, firstly, expanded our study cohort from 118 mCRC patients previously to 394 currently. Among them, 169 were *KRAS* mutant and 225 *KRAS* wild-type. Our target study population was *KRAS* mutant mCRC patients who had used and never-used oxaliplatin-based regimens. We, then, compared the difference of median OS between *KRAS* mutant mCRC patients who had used and never-used either oxaliplatin-based, irinotecan-based, bavacizumab-based, or cetuximab-based regimens, respectively. To avoid the potential bias that more drugs always result in longer OS, we also compared the difference of median OS between *KRAS* wild-type mCRC patients who had used and never-used either oxaliplatin-based, irinotecan-based, bavacizumab-based or cetuximab-based regimens, respectively. Univariate and multivariate analyses were performed in *KRAS* mutant and wild-type mCRC patients respectively to investigate whether the fact that patients who had used oxaliplatin-based regimens was an independent prognostic factor that influences the outcome only in *KRAS* mutant, but not in *KRAS* wild-type mCRC patients.

## Materials and Methods

### Patient Eligibility

Between 2007 and 2012, patients who had diagnosed as stage I to IV CRC were identified by lists obtained from Medical Information Management Office and the Cancer Registry Office of National Taiwan University Hospital (NTUH). Study subjects were further identified by the following inclusion and exclusion criteria. Patients were eligible for current study if (1) their diseases had recurred to become metastatic after an initial diagnosis of stage I to III CRC; (2) they were initially diagnosed as stage IV diseases; (3) they were older than 18 years of age; (4) they had received at least more than one month of chemotherapy with/without targeted therapy. Chemotherapy included either combination or single agent of fluoropyrimidines, irinotecan or oxaliplatin. Targeted therapy included bevacizumab or cetuximab; (5) they had adequate archival tumor samples for *KRAS* mutation analysis; (6) they had signed informed consent; (7) they had complete medical chart record and regular CT scan follow-up reports. Patients were excluded if they (1) had resectable metastatic disease and immediately received complete resection of all tumors after the diagnosis (R0 resection); (2) had multiple cancers; (3) did not use any fluoropyrimidine within their entire treatment courses; (4) had active uncontrolled infection; (5) had human immunodeficiency virus (HIV) infection; (6) had poorly controlled heart failure (New York Heart Association (NYHA) class IV). All patients were treated at NTUH, and the data for analysis was locked in December, 2012. Detailed information collected for analysis included (1) age at diagnosis, (2) sex, (3) pathology reports, (4) date that patients were diagnosed of CRC with any stages, (5) date of the disease recurred to become stage IV for those initially diagnosed of stage I to III diseases, (6) location of primary CRC, (7) sites of metastases, (8) all regimens that were used within the entire treatment courses of these patients, and (9) date of death.

### Ethics Statement

This study was approved by the institutional review board of NTUH. Patients had provided their written informed consent to participate in this study and the ethics committee of NTUH had approved this consent procedure.

### Diagnosis

Diagnosis of CRC was established by reviewing the morphology of cancer cells and immunohistochemistry (CK20 or CDX2) of pathological specimens by two independent pathologists. Disease extent was routinely determined by computed tomography (CT) of the chest, abdomen and pelvis as well as bone scans if bone metastasis was suspected. Positron Emission Tomography (PET)/CT scan was performed to identify potentially curable patients or to clarify suspected lesions to determine the clinical stage. The *KRAS* gene mutation test was performed by professionals at the pathology department in NTUH as methods described previously [7].

### Treatment

**Figure 1:**
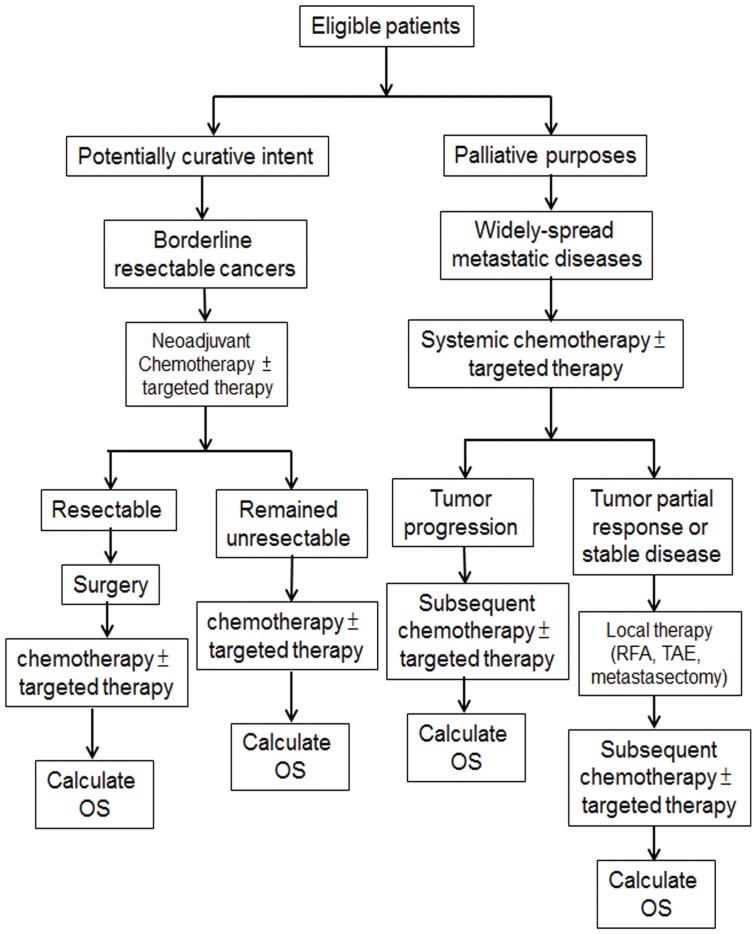
Treatment flow-chart of all the multidisciplinary treatment options that patients had received.

For patients who met all inclusion and exclusion criteria, their treatments were determined mainly by appropriate treatment goals which were administered either with potentially curative intent or for palliative purposes. Figure 1 summarized all the multidisciplinary treatment that patients had received in current study.

Patients in whom the treatment goal was potentially curative with borderline resectable cancers were treated with neoadjuvant chemotherapy with or without targeted therapy followed by surgery if applicable. In subsequently systemic treatment either for patients whose tumors had been resected or for those whose tumors remained unresectable, the same regimens were continued until the disease progressed or toxicity levels became intolerable.

Patients with widely-spread metastatic disease who were treated for palliative purposes received systemic chemotherapy with or without targeted therapy until the disease progressed or became suitable for local therapy with either radiofrequency ablation (RFA), transarterial embolization (TAE) or metastasectomy. All systemic treatments were determined by treating physicians according to patients' performance status, age, and comorbidities.

### Efficacy assessment

Median OS was assessed in *KRAS* mutant and wild-type mCRC patients. For *KRAS* mutant patients, median OS was compared between patients who had used oxaliplatin-based regimens and never-used oxaliplatin-based regimens, between patients who had used irinotecan-based regimens and never-used irinotecan-based regimens, between patients who had used bevacizumab-based regimens and never-used bevacizumab-based regimens, and, finally, between patients who had used cetuximab-based regimens and never-used cetuximab-based regimens. For *KRAS* wild-type patients, median OS was compared in the same way. Patients who had received certain given-drug-based regimens in either line of treatment within their entire stage IV treatment course was defined as patients who had used certain given-drug groups. Patients who had never received certain given-drug-based regimens in any line of treatment within their entire stage IV treatment course was defined as patient who had never-used certain given-drug groups. Median OS was calculated from the date diagnosed as stage IV CRC to the date of death, or the last visit with censoring. Patients who had not died at the time of analysis were recorded as censored at the time they were last known to be alive.

### Assessment

Tumor assessments were routinely performed by CT scan of the chest/abdomen/pelvis and by bone scan if bone metastasis was suspected at the time of diagnosis (baseline), as well as every 3 months thereafter if disease status was under control. Once the disease progression was suspected, any diagnostic tools indicated for confirmation of the progression would be performed within the 3-month interval.

### *KRAS* gene mutation assay

Seven *KRAS* mutant subtypes in codons 12 and 13 of *KRAS* exon 2 were analyzed by using DNA isolated from tumor specimens obtained from CRC patients as previously described [7].

### Statistical analysis

The chi-square test was used for the comparison of categorical variables. The Kaplan-Meier method was used to estimate median OS between *KRAS* mutant mCRC patients who had used and never-used oxaliplatin-based regimens, who had used and never-used irinotecan-based regimens, who had used and never-used bevacizumab-based regimens, and who had used and never-used cetuximab-based regimens. The same calculation was repeated again in *KRAS* wild-type patients. The log-rank test was used for univariate comparisons, and the Cox's proportional hazards model was used to identify potential prognostic factors for median OS. A *P* value <0.05 was used to indicate statistical significance; all tests were two-sided. These analyses were performed using SPSS version 16.0 for Windows.

## Results

### Patient population and treatments that patients had received

**Figure 2:**
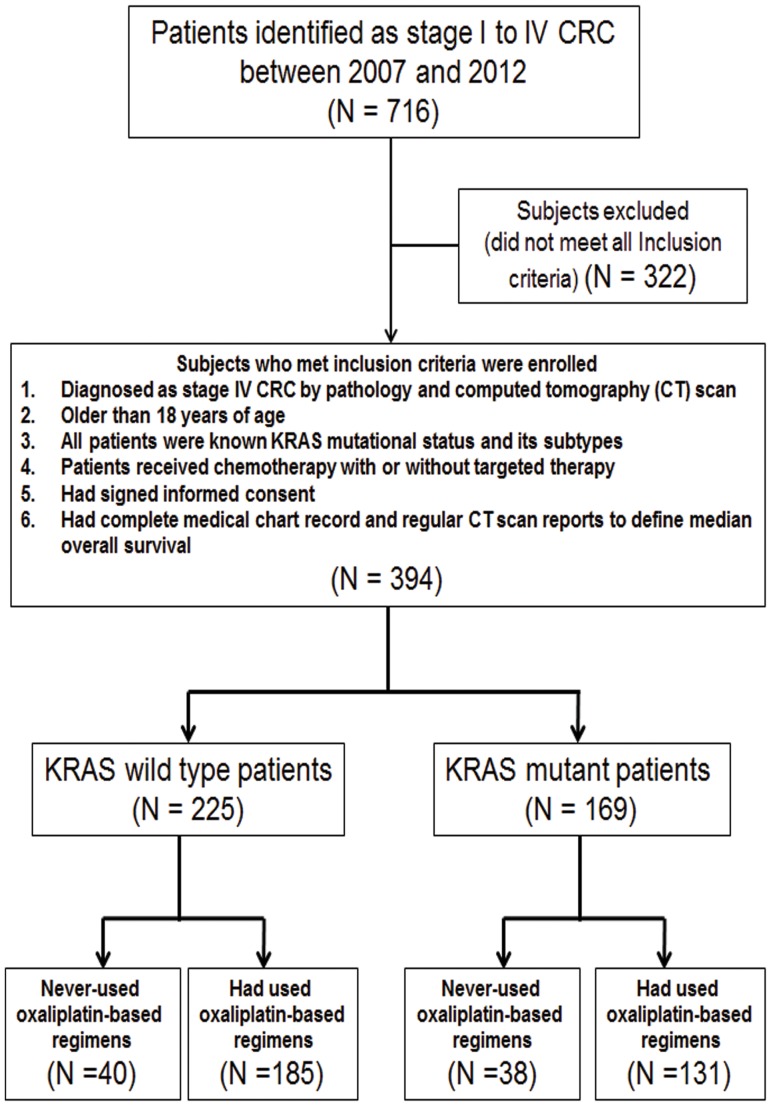
Disposition of subjects at the time of data cut-off and analysis.

Between 2007 and 2012, 716 patients who received appropriate treatment were identified as stage I to IV CRC. Of these, 394 patients were eligible to analyze in current study by inclusion and exclusion criteria. The selected patients (N = 394) were further stratified into *KRAS* mutant (N = 169) and wild-type (N = 225) groups. For both *KRAS* mutant and wild-type groups, patients were subsequently divided into those who had used and never-used certain given-drug groups. The certain given-drugs included oxaliplatin, irinotecan, bevacizumab and cetuximab. A flowchart of the patients who had used and never-used oxaliplatin-based group was shown in Figure 2. Table 1 summarized the baseline characteristics of patients who had used and never-used oxaliplatin-based regimens. Median number of lines of treatments that patients had received was shown in Table 2. The frequency of oxaliplatin-based regimens used in either locally advanced, metastatic or both stages between *KRAS* mutant and wild-type patients was demonstrated in Table 3.

**Table 1:** (*KRAS* mutant) Patient and Disease Characteristics at Baseline.

	Overall (*N* = 394)		*KRAS* mutant (*N* = 169)				*KRAS* wild type (*N* = 225)			
			Patients who had used oxaliplatin-based regimens (*N* = 131)		Never-used oxaliplatin-based regimens (*N* = 38)		Patients who had used oxaliplatin-based regimens (*N* = 185)		Never-used oxaliplatin-based regimens (*N* = 40)	
Characteristics	No. of patients	%	No. of patients	%	No. of patients	%	No. of patients	%	No. of patients	%
Sex										
Male	228	58	59	45	19	50	121	65	29	73
Female	166	42	72	55	19	50	64	35	11	27
Age, years										
Median	62		62		63		61		64	
Range	23–91		24–87		34–91		25–84		23–88	
≥65	156	40	52	40	17	45	68	37	19	48
<65	238	60	79	60	21	55	117	63	21	52
Agents had been-used										
Irinotecan	319	81	105	80	33	87	150	81	31	78
fluoropyrimidines	394	100	131	100	38	100	185	100	40	100
Cetuximab	177	45	37	28	6	16	121	65	13	33
Bevacizumab	203	52	77	59	26	68	77	42	23	58
Tumor site										
Proximal^1^	121	31	43	33	18	50	43	23	17	43
Distal^2^	273	69	88	67	18	50	142	77	23	57
No. of metastatic sites										
1	288	73	90	69	28	74	145	78	25	63
≥ 2	106	27	41	31	10	26	40	22	15	37
Initial stage										
I–III	152	39	53	40	28	74	52	28	19	48
IV	242	61	78	60	10	26	133	72	21	52

1: Proximal indicates cecum, ascending colon, hepatic flexure, and transverse colon. 2: Distal indicates splenic flexure, descending colon, sigmoid colon, and rectum.

**Table 2:** Median number of lines of treatments that patients had received.

Patients	Treatments patients received	Median lines (range)
*KRAS* mutant patients	Had used oxaliplatin-based regimens (N = 131)	2 (1–5)
	Never used oxaliplatin-based regimens (N = 38)	1 (1–4)
	Had used irinotecan-based regimens (N = 137)	2 (1–6)
	Never used irinotecan-based regimens (N = 32)	1 (1–3)
	Had used bavacizumab-based regimens (N = 103)	2 (1–6)
	Never used bevacizumab-based regimens (N = 66)	2 (1–5)
	Had used cetuximab-based regimens (N = 43)	3 (1–6)
	Never used cetuximab-based regimens (N = 126)	2 (1–5)
*KRAS* wild-type patients	Had used oxaliplatin-based regimens (N = 185)	3 (1–6)
	Never used oxaliplatin-based regimens (N = 40)	1 (1–3)
	Had used irinotecan-based regimens (N = 181)	3 (1–6)
	Never used irinotecan-based regimens (N = 44)	2 (1–3)
	Had used bevacizumab-based regimens (N = 100)	3 (1–6)
	Never used bevacizumab-based regimens (N = 125)	2 (1–5)
	Had used cetuximab-based regimens (N = 134)	3 (1–6)
	Never used cetuximab-based regimens (N = 91)	2 (1–5)

**Table 3:** The frequency of oxaliplatin-based regimens used in either locally advanced, metastatic or both stages between *KRAS* mutant and wild-type patients.

	Oxaliplatin-based regimens used in *locally advanced* stage only		Oxaliplatin-based regimens used for at least one month in *metastatic* stage only		Oxaliplatin-based regimens used in both *locally advanced* and *metastatic* stages	
*KRAS* mutant patients	19/169 (11%)	P = 0.075	116/169 (69%)	P = 0.084	15/169 (9%)	P = 0.24
*KRAS* wild-type patients	14/225 (6%)		172/225 (76%)		13/225 (6%)	

### Treatment outcomes

**Figure 3:**
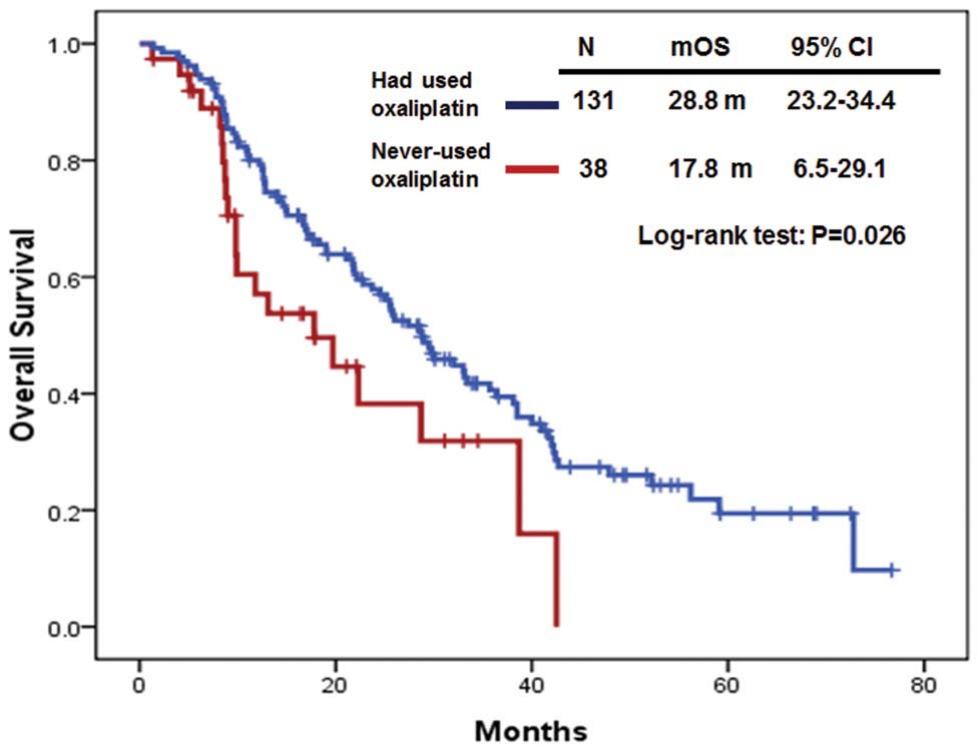
Overall survival (OS) in *KRAS* mutant mCRC patients who had used and never-used oxaliplatin-based regimens.

The median OS in *KRAS* mutant mCRC patients (N = 131) who had used oxaliplatin-based regimens was significantly longer than that in patients (N = 38) who had never-used oxaliplatin-based regimens. The median OS was 28.8 months (95% CI: 23.2–34.4) in *KRAS* mutant mCRC patients who had used oxaliplatin-based regimens and 17.8 months (95% CI: 6.5–29.1) in *KRAS* mutant mCRC patients who had never-used oxaliplatin-based regimens. (P = 0.026) (Figure 3). However, the median OS in *KRAS* mutant mCRC patients who had used either irinotecan-based, bevacizumab-based or cetuximab-based regimens was not significantly different than that in *KRAS* mutant patients who had never-used either irinotecan-based, bevacizumab-based or cetuximab-based regimens, respectively. The median OS was 28.6 months (95% CI: 23.1–34.1) in *KRAS* mutant mCRC patients who had used irinotecan-based regimens (N = 138) and 24.5 months (95% CI: 10.0–39.0) in *KRAS* mutant mCRC patients who had never-used irinotecan-based regimens (N = 31) (P = 0.71). The median OS was 22.8 months (95% CI: 13.4–32.2) in *KRAS* mutant mCRC patients who had used bevacizumab-based regimens (N = 103) and 28.7 months (95% CI: 24.3–33.1) in *KRAS* mutant mCRC patients who had never-used bevacizumab-based regimens (N = 66) (P = 0.34). Finally, the median OS was 19.0 months (95% CI: 12.4–25.6) in *KRAS* mutant mCRC patients who had used cetuximab-based regimens (N = 43) and 29.5 months (95% CI: 20.5–38.5) in *KRAS* mutant mCRC patients who had never-used cetuximab-based regimens (N = 126) (P = 0.055).

**Figure 4:**
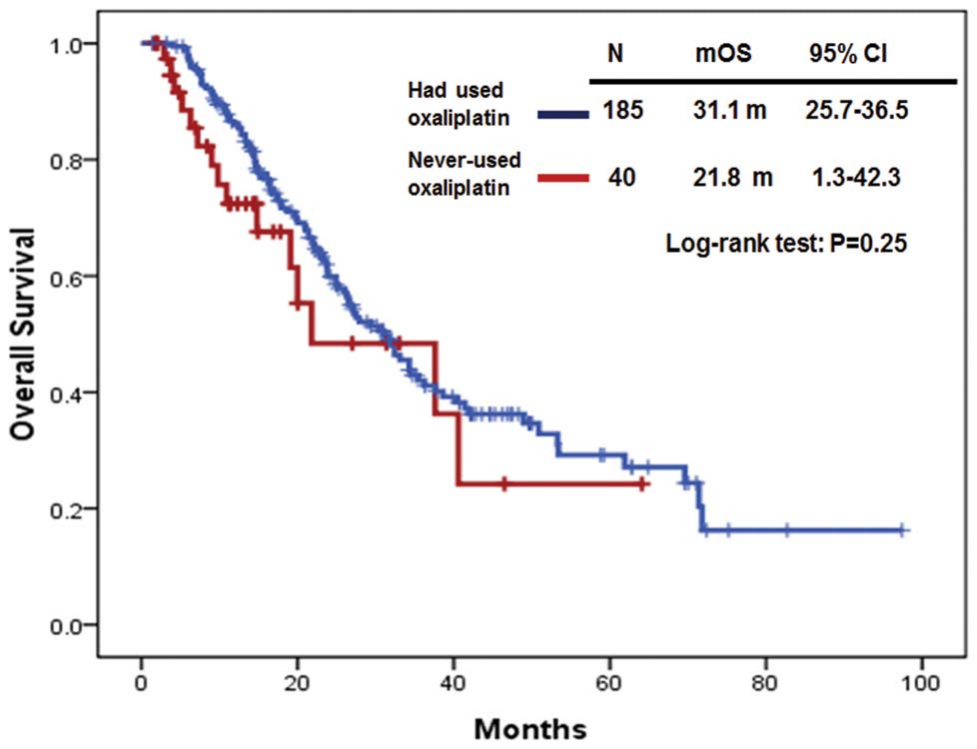
Overall survival (OS) in *KRAS* wild-type mCRC patients who had used and never-used oxaliplatin-based regimens.

On the other hand, in the group of *KRAS* wild-type mCRC patients (N = 225), the median OS in patients who had used oxaliplatin-based regimens (N = 185) was not significantly longer than that in patients who had never-used oxaliplatin-based regimens (N = 40). The median OS was 31.1 months (95% CI: 25.7–36.5) in *KRAS* wild-type mCRC patients who had used oxaliplatin-based regimens and 21.8 months (95% CI: 1.27–42.3) in *KRAS* wild-type mCRC patients who had never-used oxaliplatin-based regimens (P = 0.25) (Figure 4). In addition, the median OS was 34.3 months (95% CI: 28.2–40.4) in *KRAS* wild-type mCRC patients who had used irinotecan-based regimens (N = 181) and 15.7 months (95% CI: 12.8–18.6) in *KRAS* wild-type mCRC patients who had never-used irinotecan-based regimens (N = 44) (P<0.01). The median OS was 34.3 months (95% CI: 25.3–43.3) in *KRAS* wild-type mCRC patients who had used bevacizumab-based regimens (N = 100) and 27.2 months (95% CI: 21.0–33.4) in *KRAS* wild-type mCRC patients who had never-used bevacizumab-based regimens (N = 125) (P = 0.21). Finally, the median OS was 35.5 months (95% CI: 28.1–42.9) in *KRAS* wild-type mCRC patients who had used cetuximab-based regimens (N = 134) and 20.0 months (95% CI: 14.8–25.2) in *KRAS* wild-type mCRC patients who had never-used cetuximab-based regimens (N = 91) (P<0.01).

### Univariate and multivariate analysis

The Cox proportional hazard model was further used to test other potential confounding factors that might influence the median OS in *KRAS* mutant and wild-type mCRC patients. The factors included were age (≤65 vs >65), sex (female vs male), *KRAS* gene mutational status (codon 12 vs codon 13) in *KRAS* mutant group, initial stage of the disease (stage I–III vs stage IV), agents that patients had used within their entire stage IV treatment courses (patients who had used oxaliplatin vs never-used oxaliplatin, had used irinotecan vs never-used irinotecan, had used bevacizumab vs never-used bevacizumab, and had used cetuximab vs never-used cetuximab), oxaliplatin/fluorouracil-based adjuvant chemotherapy in stage III diseases (yes vs no), location of the tumor (distal vs proximal) and number of metastases (1 vs ≥2). In summary, in *KRAS* mutant mCRC patients, patients who had used oxaliplatin-based regimens, and only one site metastasis were independent favorable prognostic factors for longer OS which were demonstrated not only in univariate but also in multivariate analyses (Table 4). On the other hand, in *KRAS* wild-type patients, patients who had used irinotecan-based, and cetuximab-based regimens were independent favorable prognostic factors for longer OS (Table 5).

**Table 4:** Cox proportional hazard model for OS in *KRAS* mutant mCRC patients.

	Univariate analysis			Multivariate analysis		
Variable	HR^1^	95%CI^2^	P	HR	95%CI	P
Age			0.59			0.67
≥65	1.16	0.75–1.66		1.11	0.7–1.74	
<65	1.00			1.00		
Sex			0.26			0.17
Female	1.25	0.85–1.83		1.33	0.89–1.98	
Male	1.00			1.00		
KRAS			0.98			1.0
Codon 13	0.99	0.62–1.60		1.00	0.6–1.66	
Codon 12	1.00			1.00		
Initial Dx as stage IV			0.067			0.07
Yes	1.43	0.98–2.10		3.91	0.92–16.63	
No	1.00			1.00		
Primary site			0.11			0.71
Distal	0.73	0.49–1.07		0.93	0.61–1.40	
Proximal	1.00			1.00		
Cetuximab			0.057			0.17
Never-used	0.68	0.45–1.01		0.73	0.46–1.15	
Had been used	1.00			1.00		
Oxaliplatin			0.028			**0.001**
Never-used	1.73	1.06–2.82		2.45	1.44–4.15	
Had been used	1.00			1.00		
Irinotecan			0.71			0.83
Never-used	0.91	0.55–1.51		1.07	0.58–1.96	
Had been used	1.00			1.00		
Bevacizumab			0.34			0.16
Never-used	0.83	0.56–1.22		0.73	0.48–1.13	
Had been used	1.00			1.00		
Chemotherapy in stage III			0.18			0.22
No	1.30	0.89–1.92		0.4	0.95–1.72	
Yes	1.00			1.00		
No. of mets			0.005			**0.003**
1	0.56	0.38–0.84		0.54	0.36–0.81	
≥2	1.00			1.00		

1 HR: hazard ratio. ^2^CI: confidence interval.

**Table 5:** Cox proportional hazard model for OS in *KRAS* wild-type mCRC patients.

	Univariate analysis			Multivariate analysis		
Variable	HR^1^	95%CI^2^	P	HR	95%CI	P
Age			0.29			0.92
≥65	1.22	0.85–1.75		0.98	0.66–1.45	
<65	1.00			1.00		
Sex			0.97			0.74
Female	1.00	0.69–1.47		0.93	0.61–1.42	
Male	1.00			1.00		
Initial Dx as stage IV						0.14
Yes	1.10	0.74–1.63	0.63	1.94	0.81–4.65	
No	1.00			1.00		
Primary site						
Distal	0.88	0.59–1.31	0.52	0.84	0.55–1.28	0.41
Proximal	1.00			1.00		
Cetuximab			0.0001			**0.02**
Never-used	1.98	1.36–2.89		1.61	1.07–2.4	
Had been used	1.00			1.00		
Oxaliplatin						
Never-used	1.37	0.79–2.37	0.26	1.12	0.62–2.02	0.7
Had been used	1.00			1.00		
Irinotecan						
Never-used	2.49	1.63–3.83	0.0001	1.99	1.25–3.19	**0.004**
Had been used	1.00			1.00		
Bevacizumab						
Never-used	1.26	0.88–1.82	0.21	1.06	0.72–1.55	0.78
Had been used	1.00			1.00		
Chemotherapy in stage III						
No	1.02	0.67–1.54	0.93	0.54	0.22–1.36	0.18
Yes	1.00			1.00		
No. of mets						
1	0.64	0.43–0.96	0.03	0.75	0.49–1.15	0.18
≥2	1.00			1.00		

1 HR: hazard ratio. ^2^CI: confidence interval.

## Discussion

According to the current and our previously published studies, oxaliplatin-based regimens are more beneficial in *KRAS* mutant mCRC patients, because, theoretically, the earlier the effective regimens can be used, the greater probability cancers can be controlled. *KRAS* mutant mCRC patients, currently, were facing hurdles of treatment with fewer treatment options than *KRAS* wild-type mCRC patients. It is reasonable and superior to use an optimal agent first while it is currently available and has been approved on markets rather than to continuously explore new agents for which the efficacy of new agents remains uncertain. Our previously published findings and currently reported results demonstrated that oxaliplatin-based regimens in currently available treatments might result in longer first-line PFS in *KRAS* mutant than that in *KRAS* wild-type mCRC patients [7]. In addition, the median OS in *KRAS* mutant mCRC patients who had used oxaliplatin-based regimens was significantly longer than that in *KRAS* mutant mCRC patients who had never-used oxaliplatin-based regimens. To avoid the possible arguments that our current finding was resulted from selection bias and the dogma that more drugs always result in longer OS, we also validated this issue to *KRAS* wild-type mCRC patients who had used and never-used oxaliplatin-based regimens as a control group. Notably, median OS in *KRAS* wild-type mCRC patients who had used oxaliplatin-based regimens was not significantly longer than that in *KRAS* wild-type patients who had never-used oxaliplatin-based regimens. Besides, we also validated the frequency of oxaliplatin-based regimens used in either adjuvant, metastatic or both setting (Table 3) and measured median time to recurrence from locally advanced (stage II or III) to metastatic setting (Table 6) between *KRAS* mutant and wild-type patients to ensure all of these parameters between these two groups calculated in our current study were relatively balanced. Furthermore, median OS in *KRAS* mutant patients who had used either irinotecan-based, bevacizumab-base or cetuximab-based regimens was not consistently and significantly longer than that in *KRAS* mutant patients who had never-used either irinotecan-based, bevacizumab-base or cetuximab-based regimens. These findings further strengthened and highlighted the potential crucial role of oxaliplatin in *KRAS* mutant mCRC.

**Table 6:** Median time to recurrence (TTR) and the percentage of TTR less and more than 6 months after adjuvant oxaliplatin-based regimens between *KRAS* mutant and wild-type patients.

	Median time to recurrence		TTR less than 6 months after oxaliplatin-based regimens	TTR longer than 6 months after oxaliplatin-based regimens	
*KRAS* mutant patients	11.4 mons (8.1–14.7)	P = 0.67	10/34 (29%)	24/34 (71%)	P = 0.53
*KRAS* wild-type patients	9.2 mons (7.0–11.4)		10/27 (37%)	17/27 (63%)	

The results of our current study let us move one step forward toward our major research theme that oxaliplatin indeed is crucial in *KRAS* mutant mCRC patients although prospective randomized clinical study remains warranted to definitely conclude the results. However, to the best of our knowledge, in the clinical circumstance to evaluate whether a certain given treatment is better than another, the objective study-end-points are usually set as first-line PFS or OS [8]–[10] which were just like those set in our serial studies. We have previously demonstrated that first-line PFS in *KRAS* mutant mCRC patients was significantly longer than that in *KRAS* wild-type mCRC patients. This time, we further demonstrated oxaliplatin was indeed crucial in *KRAS* mutant mCRC patients by proving that longer OS was observed in *KRAS* mutant patients who had used oxaliplatin-based regimens compared to OS in *KRAS* mutant patients who had never-used oxaliplatin-based regimens. Other potential bias that might confound our study results was also adjusted by multivariate analysis in current study. Notably, an Italian group recently published a study [11] which was designed and reported similarly to our previously published study [7]. Their results additionally stated that median OS was significantly longer in *KRAS* mutant mCRC patients than that in *KRAS* wild-type patients when both of them received oxaliplatin-based regimens. Their report echoed with our serial research findings.

In pre-targeted therapy era, Tournigand et. al. [12] published a pivotal article reporting that first-line chemotherapy with either irinotecan/5FU/lecovorin (FOLFIRI) or oxaliplatin/5FU/leucovorin (FOLFOX6) in “non-selected” mCRC patients did not influence OS. Both regimens could thus be recommended as first-line treatment for mCRC. In post-targeted therapy era, our recently published work demonstrated that *KRAS* gene mutation is not only an insensitive biomarker to EGFR monoclonal antibodies but also a sensitive predictor to oxaliplatin-based regimens in personalized chemotherapy of mCRC treatment.

Although EGFR monoclonal antibodies are not suggested to use in *KRAS* mutant mCRC patients [5], our study cohort did have some patients (25%) who had received cetuximab-based regimens within their treatment course. The reasons for this deviated treatment were usually attribute to that those patients who had their treatment before the consensus that *KRAS* mutant mCRC patients were not recommended to use EGFR monoclonal antibodies. In addition, panitumumab has never been available in Taiwan, therefore, in our study cohort, we did not have patients who had used panitumumab-based regimens.

Multivariate analysis in the present study revealed that in *KRAS* mutant mCRC patients, except for the variable that patients who had used oxaliplatin-based regimens which was what we were the most interested in, only one site metastasis was the other independent favorable prognostic factors for longer OS. At the meantime, for *KRAS* wild-type mCRC patients, variables of patients who had used irinotecan-based and cetuximab-based regimens were independent favorable prognostic factors for longer OS. All of these findings were reasonable and may repeatedly confirm the quality and accuracy of the data.

There are still some limitations in this retrospective study which include the uneven number of patients (*KRAS* wild-type and mutant) who had or had not used oxaliplatin-based regimens and that we cannot fully extrapolate the synergy effect of oxaliplatin in combination with other chemotherapy agents and biologic agents in this analysis, although we have already done our best to overcome selection bias in this study by dissecting the subgroups and performing multivariate analysis. The limitations and results we report in this study are reasons to conduct a prospective study to validate our findings in the future.

In conclusion, our data suggests that oxaliplatin-based chemotherapy is more beneficial in *KRAS* mutant mCRC patients than in *KRAS* wild-type mCRC patients.
